# Cancer-associated fibroblast infiltration in gastric cancer: the discrepancy in subtypes pathways and immunosuppression

**DOI:** 10.1186/s12967-021-03012-z

**Published:** 2021-07-31

**Authors:** Xu Liu, Li Yao, Jingkun Qu, Lin Liu, Ning Lu, Jiansheng Wang, Jia Zhang

**Affiliations:** 1 Department of Thoracic Surgery, The First Affiliated Hospital of Xi’an Jiaotong University, 277 West Yanta Road, Xi’an, Shaanxi, China; 2 Department of Neurology, Xi’an XD Group Hospital, 97 North Fengdeng Road, Xi’an, Shaanxi China; 3 Department of Oncology, The Second Affiliated Hospital of Xi’an Jiaotong University, 157 West Fifth Street, Xi’an, Shaanxi China; 4 Department of Gastroenterology, The First Affiliated Hospital of Xi’an Medical University, 48 West Fenghao Road, Xi’an, Shaanxi China

**Keywords:** Gastric cancer, Fibroblast, Prognostic, Subtype, Immunosuppressive

## Abstract

**Background:**

General role of cancer-associated fibroblast (CAF) and its infiltration characteristics in gastric cancer remains to be unknown.

**Methods:**

We estimate CAF infiltration in bulk tumor tissue with RNA-seq data and analyzed its relationship with gastric cancer subtype, survival and immune microenvironment.

**Results:**

We revealed CAF intend to have higher infiltration in diffuse, genomically stable, and advanced gastric cancer. CAF is associated with immunosuppressive microenvironment. Wide transcriptomics alterations occur in high CAF infiltrated gastric cancer, PI3K/AKT, TGFB and Hedgehog pathway are remarkable in this procedure. We utilized receptor tyrosine kinases and TGFB pathway ligands to construct risk score system that can predict survival.

**Conclusion:**

Thus, CAF is associated with aggressive phenotype of gastric cancer and risk score based on RTK and TGFB pathway ligands expression is a promising tool for assessment of gastric cancer survival.

**Supplementary Information:**

The online version contains supplementary material available at 10.1186/s12967-021-03012-z.

## Introduction

Gastric cancer (GC) is the fifth most common cancer globally. In 2018, gastric cancer-caused death ranks the third among all kinds of cancer [1]. Although therapeutics including surgical technology, radiotherapy, neoadjuvant chemotherapy has undergone significant development, the 5-year survival is continuously unsatisfying [2]. Multiple interactions of genetic, environmental and host factors brings tremendous complexity and heterogeneity to gastric cancer [3]. Several classification systems were developed, such as Lauren classification, WHO classification and The Cancer Genome Atlas (TCGA) subtype, striving to managing this heterogenous cancer as fine as possible [3].

The tumor microenvironment(TME) is defined as the complicated eco-system within bulk tumor tissue comprising of multicellular and stroma component including immune cells such as T and B lymphocytes, dendritic cells (DC), natural killer (NK) cells, tumor-associated macrophages (TAM), neutrophils, and myeloid-derived suppressor cells (MDSC) cancer-associated fibroblasts (CAF) [4]. Under the circumstances of immune-therapy entering into clinical application widely, vast majority of studies focusing on understanding the role of TME component have emerged to assist improving immune-therapy efficiency. CAF a major part of stroma cell that produces extracellular matrix (ECM) not only promotes tumor growth and invasion through secreting all varieties of cytokines, exosomes, and growth factors, but also creates immunosuppressive TME in several types of cancer [4, 5]. Controversially, some studies demonstrated depletion of CAF activation signaling is in favor of tumor progression and certain CAF subpopulation could also inhibit tumor growth and metastasis implying CAF’s tumor-restrictive role [6].

In gastric cancer, studying on the role of CAF remains to be largely marginal. Here, we estimated CAF infiltration in several large-sample gastric cancer cohorts by utilizing three published bioinformatic algorithms, MCPCOUNTER [7], XCELL [8], EPIC [9] and analyzed the correlation of CAF infiltration in bulk tumor tissue to clinical, transcriptomics, proteomic and immune micro-environment characteristics. Benchmark analysis demonstrate CAF score estimated by all these three method exhibited collinearity with CAF proportion (r > 0.72) in simulated bulk sample from single cell RNA-seq data [10]. We found that CAF is highly infiltrated in diffuse (DGC) and genomically stable (GS) gastric cancer. Higher CAF infiltration is also related to III/IV stage disease condition. To our knowledge, this is the first study issuing CAF is differentially infiltrated between different Lauren and TCGA subtype. Previous sporadic study in which CAF were counted by IHC staining of CAF markers like α-SMA or FAP demonstrated high CAF infiltration in gastric cancer indicates poor survival [11–13]. We validated this conclusion in multiple datasets. We revealed wide pathway and transcriptional programing alteration in high CAF infiltration micro-environment. We also first uncovered CAF might be associated with immunosuppressive micro-environment in gastric cancer tissue. Finally, we established risk model based on key pathways ligands TGFB2, VEGFB, COL10A1, AREG and EFNA5 to predict gastric cancer survival. Comprehensively, this study gave a multi perspective functional landscape of CAF and shed light on its role in gastric cancer.

## Materials and methods

### Data acquisition

Regarding to TCGA STAD dataset, RNA-seq, miRNA expression and clinical data was acquired from UCSC Xena portal (https://xenabrowser.net/datapages/). Homologous recombination deficiency (HRD) score deposited in Pan-Cancer Atlas Hub was also extracted from UCSC Xena. TCGA DNA Damage Repair Analysis Working Group (DDR-AWG) calculated this score by using somatic copy number alteration (SCNA) calls generated from ABSOLUTE [14]. Briefly the degree of three different forms of genomic scars: LST (large-scale state transitions) [15], the loss of heterozygosity (LOH) [16] score and NtAI (number of telomeric allelic imbalances) [17] scores were estimated by SCNA data via the published algorithms, the HRD score is defined as the sum of these three score to reflect genomic instability caused by homologous recombination deficiency. The Reverse Phase Protein Array (RPPA) data was from cBioPortal (http://www.cbioportal.org/). MSI score and aneuploidy score for each sample has already precalculated by cBioPortal referring Niu [18, 19] and Talor’s [20] PanCancer study, respectively. GSE15459 (192 cases), GSE84437 (434 cases), GSE62254 (300 cases), GSE26901 (110 cases), GSE26253 (433 cases) gene expressional array data with clinical information was downloaded from Gene Expression Omnibus database of NCBI (https://www.ncbi.nlm.nih.gov/gds/).

### Assessment of CAF and immune cells infiltration

Quantification of cell component inside tumor tissue was evaluated with TIMER2.0 online-tool (http://timer.cistrome.org/) that integrates six algorithms, including TIMER, xCell, MCP-counter, CIBERSORT, EPIC and quanTIseq Gene [21]. CAF level was estimated by three algorithms: MCPCOUNTER [7], XCELL [8], EPIC [9]. The whole estimation result matrix can be received directly after expression matrix annotated with gene symbol being uploaded onto the TIMER2.0 server.

### Differentially expressed gene (DEGs) analysis

Samples were categorized into three groups according to CAF infiltration level estimated by the three methods mentioned above. First of all, we ranked these three CAF scores, trisected all the samples in each cohort and labeled with “high”, “medium”, “low”. To mitigate categorical error that each estimation method might bring, we define samples that were classified into high group by at least two method as consensually high group. Similarly, samples fall into low group assessed by at least two method were assigned into consensually low CAF group. Otherwise, the left samples were regarded as medium/unsure CAF group. Then, DEGs between high and low CAF group were analyzed by "limma" package (Ver. 3.46.0) in software with normalized gene or miRNA expression data.

### Gene set enrichment analysis (GSEA)

C2 curated gene sets which collects canonical pathways was download from MSigDB for GSEA analysis. Among all the DEG, genes with adjust P value < 0.05 were taken into GSEA analysis by GSEA function of “clusterProfiler” package (Ver. 3.18.0) of R [22]. Gene sets of which P value < 0.01 and FDR q value < 0.05 were regarded as significantly enriched term.

### Statistical analysis

All the graphic and statistical work was accomplished by ORIGIN software or R program (Ver. 4.0.3). Two-group comparisons were conducted by Mann–Whitney test, multigroup analysis were performed by using ANOVA. R package “survival” (Ver. 3.2-7)”, survminer” (Ver. 0.4.9) [23, 24] were utilized to perform survival analysis. To establish prognostic risk score model, we screened candidate genes by two steps: we first performed univariate cox analysis. Then, genes that were statistically associated with overall or disease-free survival were included for lasso cox regression run by “glmnet” package (Ver. 4.1-1) to generate candidate list. After all, genes which were present in lasso model at minimum lambda value were used to develop risk score system by multivariate COX regression. Once the COX model together with coefficient for each gene were obtained, risk score can be calculated by following formula:

$$Risk\, Score= \sum coefficient\left(gene i\right)*expression\,value(gene i).$$

## Results

### CAF intends to be higher infiltrated in diffused, genomically stable and late stage gastric cancer

**Fig. 1:**
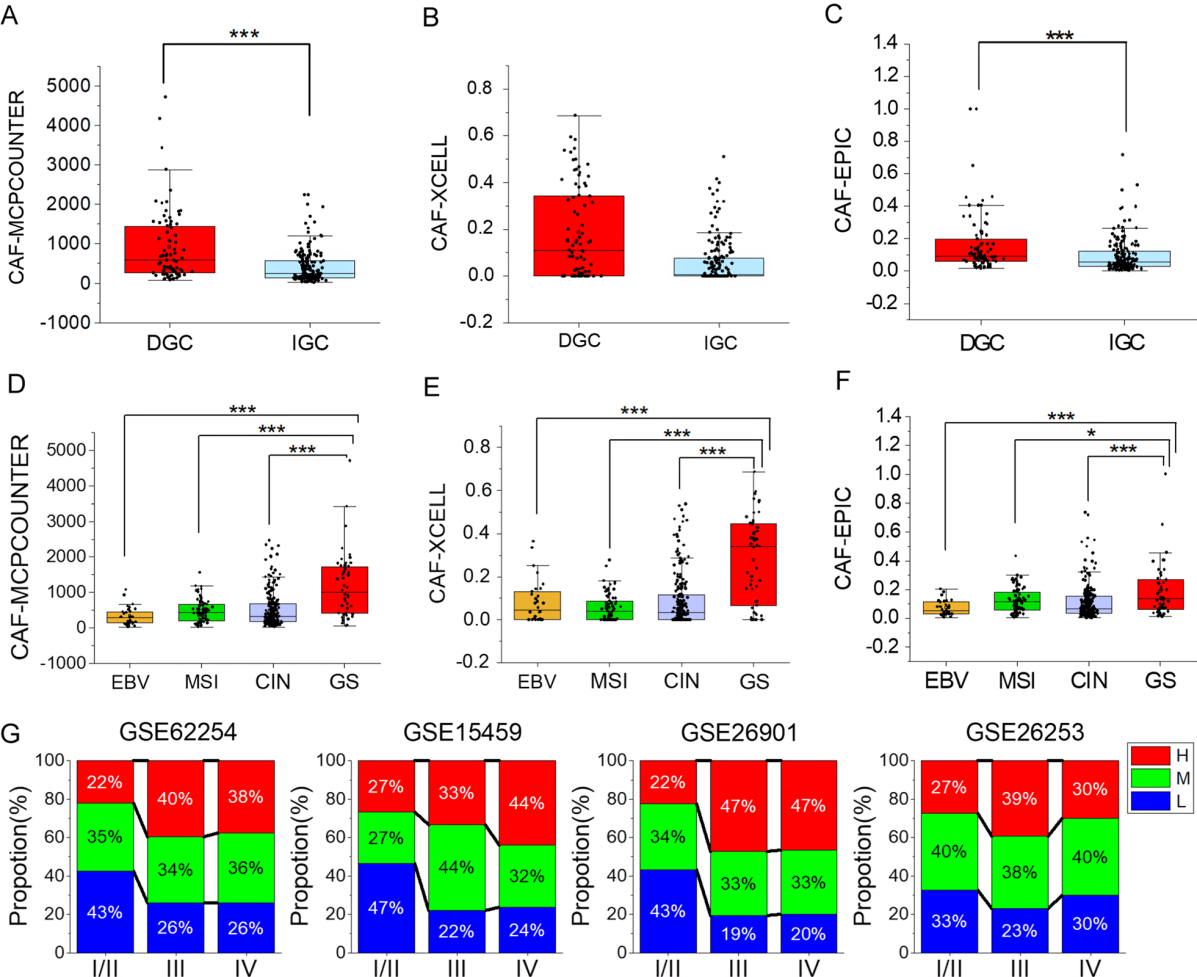
CAF infiltration across Lauren subtypes, TCGA molecular subtypes and pathological stages. Differential infiltration of CAF in intestinal and diffuse type of gastric cancer in TCGA cohort, CAF was estimated by MCP-COUNTER (**A**), XCELL (**B**) and EPIC (**C**). **D**–**F** Differential infiltration of CAF in EBV(+), MSI, CIN and GS tumor in TCGA cohort, CAF was estimated by MCP-COUNTER (**D**), XCELL (**E**) and EPIC (**F**). **G** Percentage of high (H), medium (M), and low (L) CAF infiltration in different pathological stages. *P < 0.05, **P < 0.001, ***P < 0.0001

Among the versatile classification systems, Lauren classification which divides gastric cancer into intestinal subtype (IGC)and diffuse type (DGC) is commonly used as it can better clusters gastric cancer with similar tumor biological characteristics together [25, 26]. To explore whether different type of gastric cancer possesses distinct CAF infiltration, we extracted diffuse, signet ring and intestinal stomach adenocarcinoma samples in TCGA cohort and compared their CAF score assessed by three algorisms. We attribute signet ring carcinoma into diffuse type for its poorly cohesive and submucosally invasive properties [27]. Attractively, general CAF quantification in DGC group is more abundant than IGC with a statistically significant level (Fig. 1A–C), even though there exists overlapped CAF level between the groups. We validated this in another cohorts GSE15459, GSE26901, GSE26253, GSE62254. As described in method section, three-algorithm based CAF consensus grouping were performed. A larger percentage of samples with high and medium fibroblasts content were observed to be distributed in DGC in GSE15459, GSE26253 and GSE62254 datasets (Additional file 5: Fig. S1A). Thus, intra-tumoral fibroblast is differentially infiltrated between two Lauren subtypes.

We further checked CAF in different TCGA subtypes including Genomically stable (GS), Chromosomal instability (CIN), Microsatellite Instable (MSI) and EBV(+) [28]. Amazingly, GS subtype exhibits significantly higher abundance of fibroblasts than CIN, MSI and EBV (+) gastric cancer. Meanwhile, fibroblasts quantification in CIN subtype accountable for more than half stomach adenocarcinoma in TCGA cohort varies vastly and some CIN patients can have extremely high CAF infiltration as well (Fig. 1D–F).

Next, we analyzed the variation in CAF level with cancer stage. Unfortunately, we failed to detect remarkable difference for fibroblasts counts in different AJCC stages. However, the percentages of patients with high and medium CAF infiltration in stage III and IV (Fig. 1G) increases dramatically suggesting fibroblast infiltration might be involved in gastric cancer progression. Considering unbalanced CAF enrichment in GS tumor, we specially investigated CAF infiltration discrepancy in stage III and IV tumors to disentangle the impact of molecular subtype and disease staging. Fibroblast in GS tumor remains to be the highest across four molecular subtypes even when only taking stage III/IV samples into consideration (Additional file 5: Fig. S1B). Concurrently, GS tumors distributing in 20 of 165 stage I/II samples and 28 of 201 stage III/IV samples proposes GS tumor is not associated with disease stage and the biased infiltration of CAF in GS tumor is independent on the later one.

### CAF infiltration is related to deteriorated survival for gastric cancer

In spite of numerous studies indicating CAFs’ tumor-fostering effects, some also asserted certain CAFs subpopulation inhibit tumor progression [6]. Thus, we retrospectively inspected comprehensive function of CAF estimated by MCPCOUNTER, XCELL and EPIC in gastric cancer prognosis. All of the three methods compute immune and stroma cell abundance relying on transcriptomics signature. They use three very distinct algorithms: Geometric mean of expression of marker genes, single-sample GSEA (ssGSEA) and constrained least square regression to estimate cell population, respectively [29]. Among them, only the cell abundance score by EPIC can be interpreted as cell fraction. Nevertheless, the scores calculated by each of them are comparable among samples. We performed survival analysis to explore prognostic indication by single CAF estimation method. Only MCPCOUNTER can discriminate the overall survival of high CAF patients with low CAF in five cohorts whereas, stratification by the other two do not always work well in five cohorts we collected (Additional file 1).

**Fig. 2:**
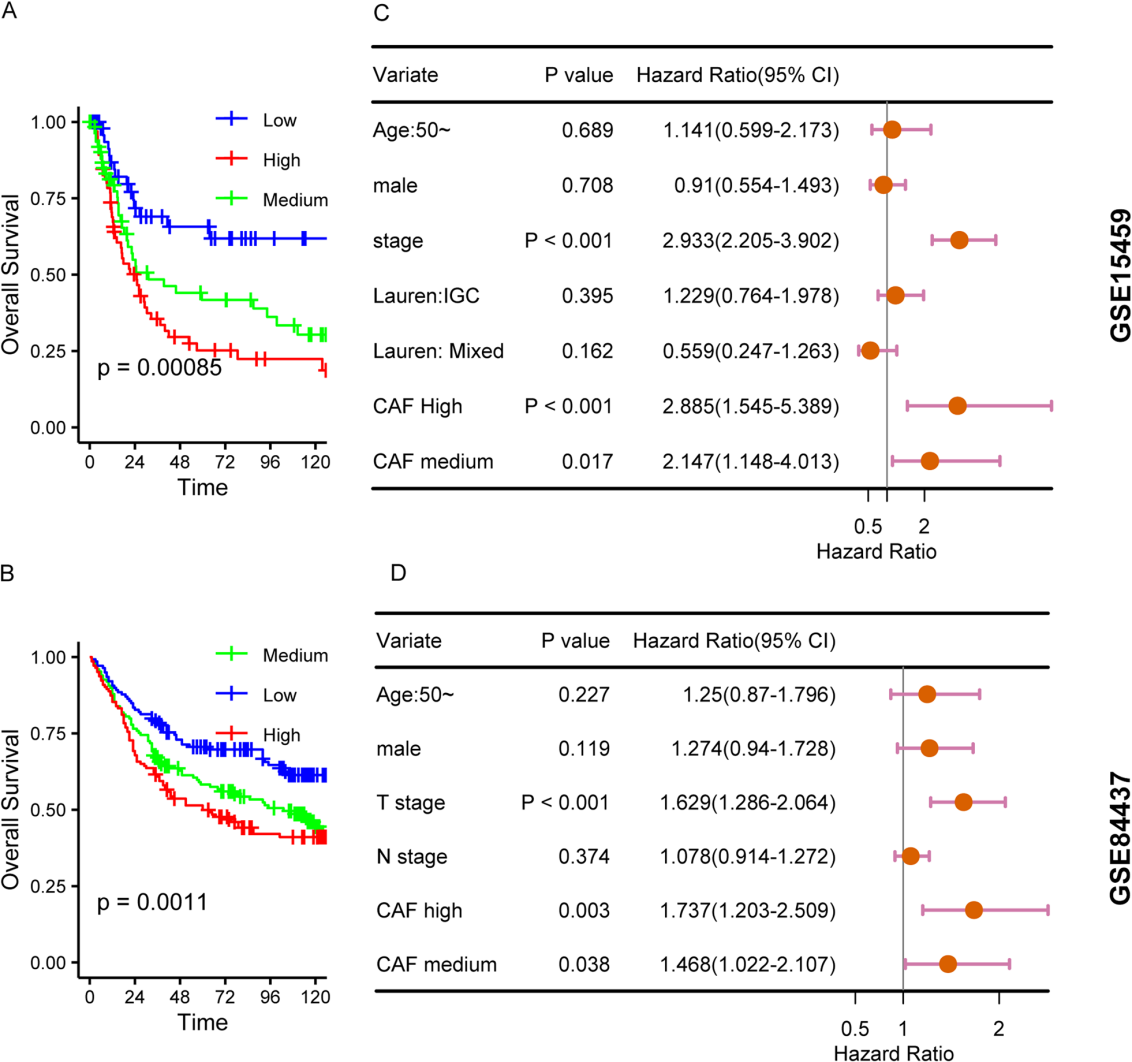
CAF infiltration and Overall survival in GSE15459 and GSE84437 cohorts. **A** Kaplan–Meier plot of CAF high, medium and low group in GSE15459 cohort. **B** multivariate COX regression analysis of overall survival in GSE15459 cohort. **C** Kaplan–Meier plot of CAF high, medium and low group in GSE84437 cohort; **D** multivariate COX regression analysis of overall survival in GSE84437 cohort

We then used combined CAF stratification by all these three methods to scrutinize the performance of CAF in gastric cancer survival. We included 239 TCGA samples provided both overall (OS) and disease-free survival (DFS) information for survival analysis (Table1). Univariate cox analysis showed Hazard ration (HR) of high CAF group for OS is 2.27 (CI = 1.09–4.73, P = 0.0285), and HR for DFS is 2.26(CI = 1.04–4.91, P = 0.0398). HR for OS in multivariate COX analysis is 2.12(CI = 1.04–4.71, P = 0.0391). We used GSE15459, GSE84437, GSE26901, GSE26253 and GSE62254 datasets to further validate this impact. As expected, the overall survival of high CAF group can be discerned well from CAF low group, (Fig. 2A, C; Additional file 6: Fig. S2A–C). In GSE62254, GSE26901, GSE26253, three cohorts with DFS data, high CAF group exhibits shorter disease-free survival time (Additional file 6: Fig. S2D–F). To rule out the confounding effect caused by Lauren classification and tumor stage, multivariate COX analysis was performed and the results revealed CAF infiltration is an independent factor for gastric cancer survival (Fig. 2B, D). Take all these data into consideration, we suggest MCPCOUNTER, XCELL, EPIC based CAF estimation could be a promising strategy for prognostic assessment in gastric cancer.

**Table1:** Clinical characteristics CAF and gastric cancer survival in TCGA cohort

Variates		Cases	Univariate HR 95% CI (P Value)		Multivariate HR 95%CI (P value)	
			OS	DFS	OS	DFS
Age	< 50(ref)	20				
	50	219	0.5409–4.15 (0.437)	0.35–2.25 (0.798)	0.55–4.39 (0.4116)	0.35–2.33 (0.8790)
sex	Female(ref)	94				
	male	145	0.68–2.03 (0.562)	0.98–3.87 (0.0557)	0.67–2.03 (0.5917)	1.02–4.05 (0.0440)
Lauren classification	Intestinal(ref)	99				
	Diffuse	44	0.61–2.57 (0.538)	0.80–4.18 (0.1686)	0.52–2.35 (0.7959)	0.65–3.76 (0.3134)
	NOS	95	0.93–3.03 (0.088)	1.17–4.86 (0.0163)	0.81–2.80 (0.1966)	1.07–4.73 (0.0329)
Stage	I/II(ref)	138				
	III	101	0.95–2.71 (0.0783)	0.58–1.91 (0.866)	1.02–3.00 (0.0420)	0.62–2.06 (0.7085)
CAF	Low(ref)	73				
	Medium	81	1.04–4.66 (0.0389)	0.67–3.66 (0.3037)	0.97–4.57 (0.0591)	0.54–3.10 (0.5632)
	High	85	1.09–4.73 (0.0285)	1.04–4.91 (0.0398)	1.04–4.71 (0.0391)	0.87–4.24 (0.1081)

*OS* overall survival, *DFS* disease-free survival, *95%CI*: 95% confidence interval

### Wide transcriptomic and pathway alteration in CAF excessively infiltrated micro-environment

**Fig. 3:**
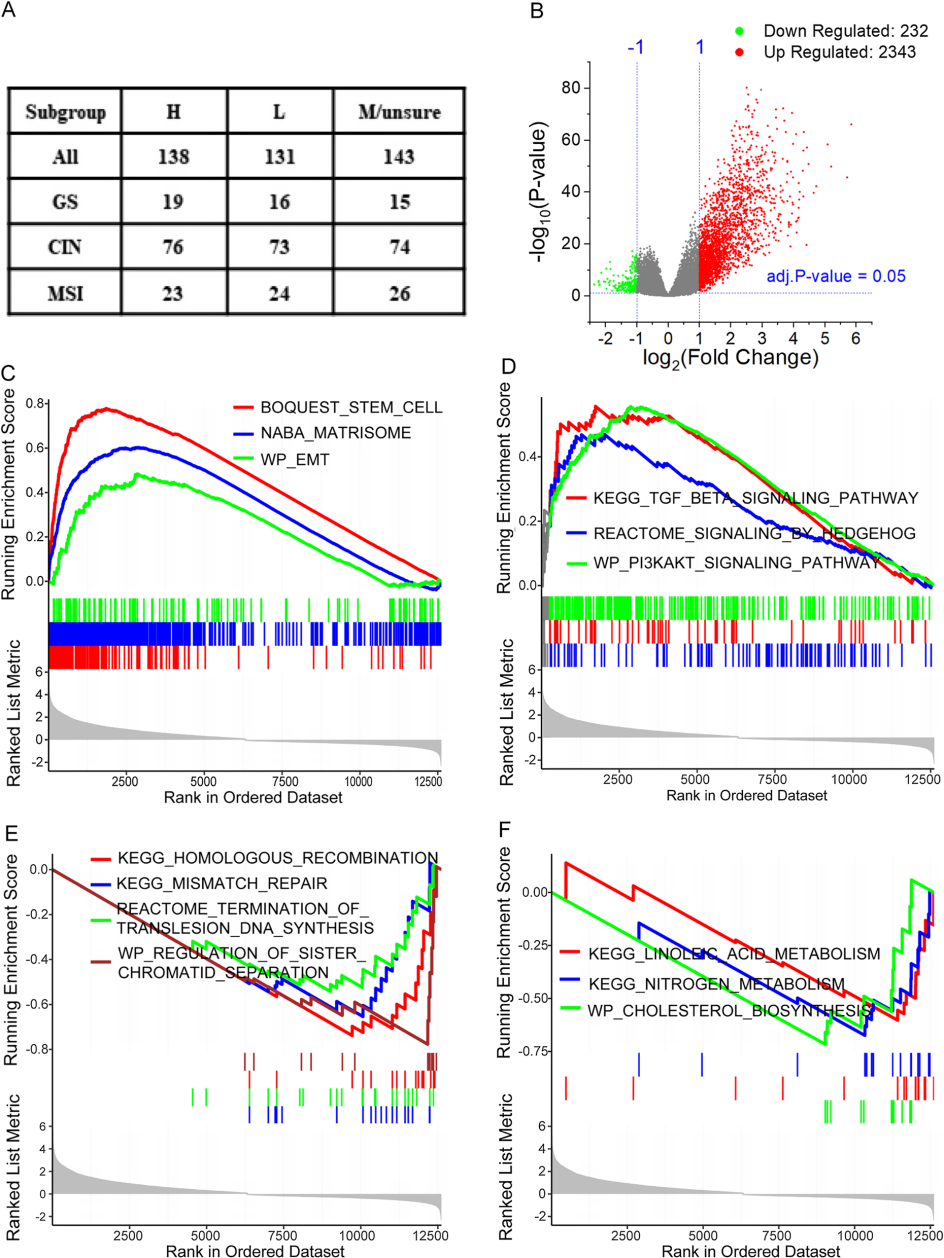
DEG analysis between CAF high and low group and GSEA reults in TCGA cohort. **A** Case number that were categorized into CAF high (H), medium (M/unsure), and low (L) group. **B** Volcano plot that displays the DEGs comparing CAF high with low group. **C**–**F** GSEA analysis showed key biological processes, and pathways involved in DEGs

To systematically explore how gene expression profile in gastric cancer tissue is shaped by CAF infiltration, we performed differential gene-expression analysis in TCGA cohort comparing mRNA expression of 138 cases of high CAF samples and 131 low CAF samples (Fig. 3A). Under the criteria of |logFC|≥ 1 and adj.P value < 0.05, 2343 genes were upregulated while only 232 were downregulated (Fig. 3B) suggesting gene expression is more likely to be transcriptionally activated than being repressed in CAF excessively infiltrated tumor tissue. Furtherly, we performed Gene Set Enrichment Analysis(GSEA) to search what biological pathway or functional genes are dysregulated under CAF highly infiltrated condition. The result manifested broad gene sets expressional differences between high and low CAF group. Matrisome composed of core ECM protein, ECM-associated proteins, ECM-affiliated proteins and secreted factors [30] are enriched in CAF highly infiltrated group. Stem cell and epithelial-to-mesenchymal transition (EMT) gene sets are also enriched in high CAF group (Fig. 3C). Regarding to pathway alterations, we observed an enrichment of PI3K-AKT and HEDGEHOG pathway gene in CAF higher group (Fig. 3D). In line with enrichment of EMT associated gene sets, TGFB pathway genes are significantly upregulated in samples with more fibroblast. Interestingly, we found that DNA damage response and repair gene sets including base excision repair, mismatch repair, trans-lesion DNA synthesis and homologous recombination, the several common used DNA repair mechanisms are enriched in CAF low group (Fig. 3E, Additional file 1). Moreover, enrichment of gene sets related to cell cycle, DNA replication, and sister chromosomal separation during mitosis are observed in lower CAF group. In addition, several metabolic related gene sets including cholesterol synthesis and nitrogen metabolism are enriched in barren CAF group (Fig. 3F).

Even though the enrichment of DNA damage and repair gene in low CAF group, there is a possibility that these genes might be upregulated responsively when DNA damage occurred because of either exogenous or endogenous reasons. Endogenous reason includes deficiency in repair mechanism such as mismatch repair which causes MSI characterized as abnormal insertions or deletions of microsatellite sequences [31] and homologous recombination deficiency(HRD) which might result in chromosomal instability [32]. As CIN and MSI tumor have relatively lower fibroblast score in our prior results (Fig. 1D–F), to ruled out the GSEA results confounding by TCGA subtype, we reperformed our DEG and GSEA analysis in these three subtype: GS, CIN and MSI respectively (Fig. 3A) and obtained consistent results (Additional file 2). Similar result was yielded when extending our analysis to GSE62254 datasets (Additional file 3). These results revealed multiple tumor biological processes and pathways are involved in CAF infiltrated microenvironment.

### CAF accumulation in gastric cancer tissue is inversely associated with severity of genomic alteration

**Fig. 4:**
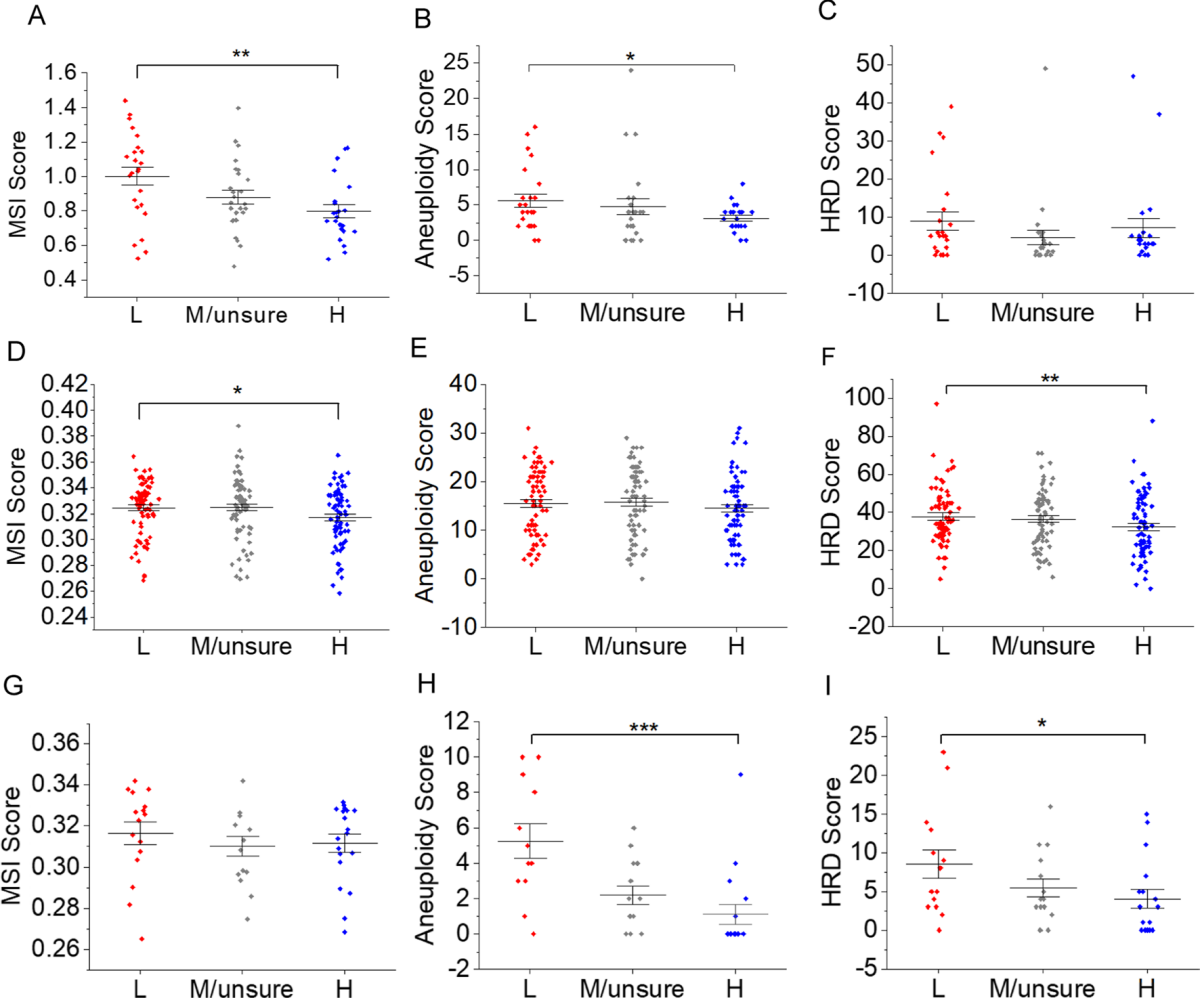
Comparing of MSI, aneuploidy, and HRD score in CAF high (H), medium (M/unsure), and low (L) group. **A**–**C** MSI, aneuploidy, and HRD score in MSI tumor. **D**–**F** MSI, aneuploidy, and HRD score in CIN tumor. **G**–**I** MSI, aneuploidy, and HRD score in GS tumor

In our GSEA analysis, we reported DNA repair genes are enriched in gastric cancer with less CAF resident without affecting by TCGA subtype. Even in GS tumor, there exists expressional difference of DNA damage repair genes when compared low CAF samples with the high. For the reason of tumor heterogeneity, we propose that in GS subtype, there exists certain degree of DNA damage or genomic instability. Similarly, the severity of DNA damage or genomic alteration in CIN and MSI tumor could vary although the samples were categorized to the same subtype. Meanwhile, the relatively mild fibroblasts accumulation in MSI and a part of CIN subtype tumor forced us to considering whether CAF and DNA damage/genomic alteration in tumor are exclusive to each other in rather heterogenous tumor tissue. Alternatively speaking, the presence of one between these two could hinder the other one accumulating in tumor tissue during tumor evolution. To validate our concern, we compared the difference of MSI, HRD and aneuploidy score, three well-established parameters to reflect extent of MSI, HRD and change of karyotype in bulk tumor tissue by other researchers [14, 18–20] at different fibroblast level in GS, CIN and MSI tumor, respectively. What need to be mentioned is HRD is not the sole mechanism for CIN. Aneuploidy, another form of CIN caused by inappropriate chromosomal segregation and manifesting as aberrated chromosomal number is another dominant [33]. As expected, in MSI cancer, the average level of MSI and aneuploidy score for low CAF group are statistically higher than that in high CAF group (Fig. 4A, B). In GS tumor, the aneuploidy and HRD score of low CAF group have an advantage over high CAF group (Fig. 4H, I). Although the discriminability of MSI and HRD score between high and low CAF group in CIN tumor is not very good, we still observed statistically significant differences (Fig. 4D, F). Taking all these results into consideration, we conclude that fibroblasts and genomic alteration in gastric cancer might accumulate towards an opposite direction.

### Differential proteomic and mi-RNA expression in varied CAF context

**Fig. 5:**
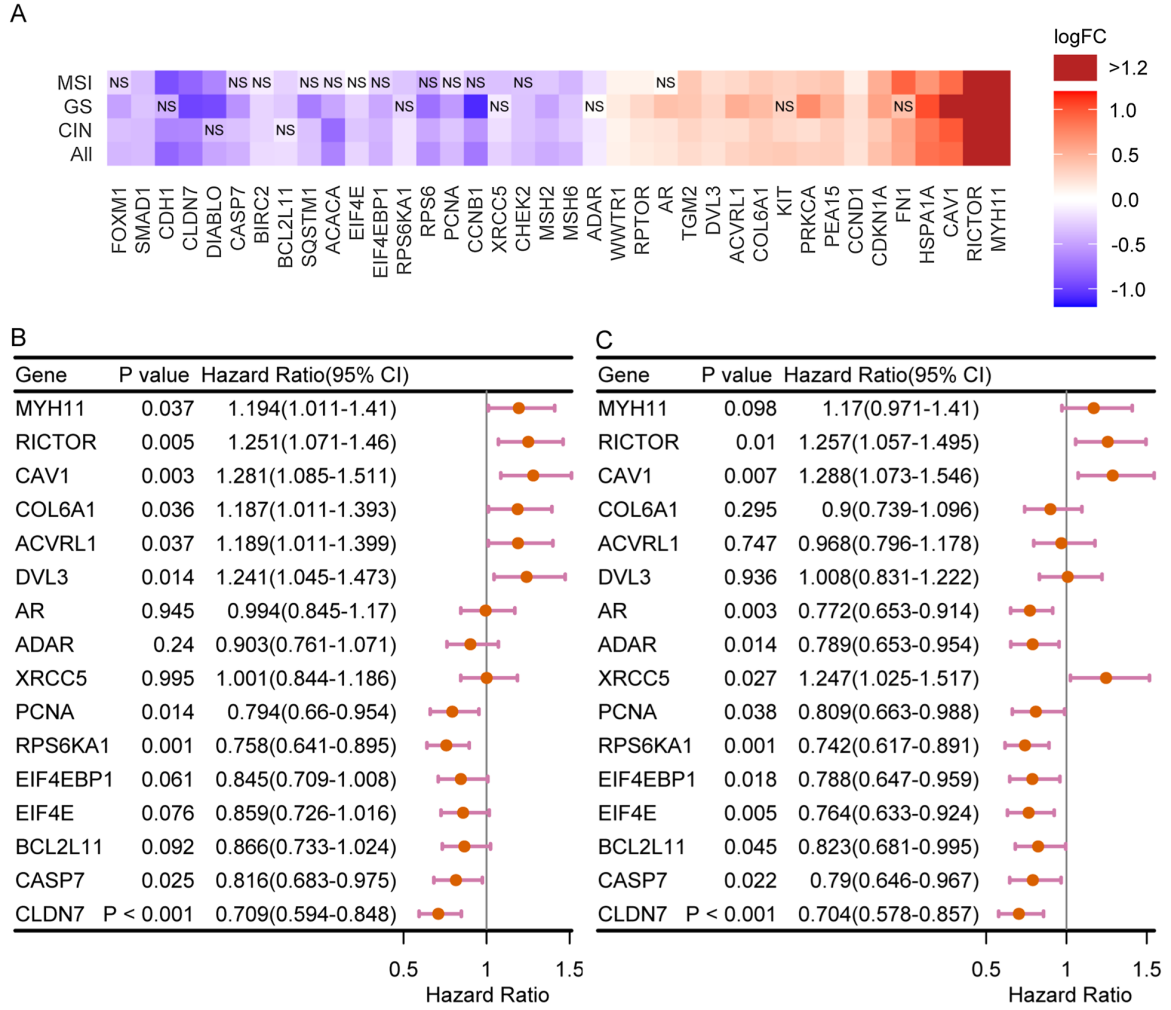
Proteomics data of different CAF infiltration. **A** Differential protein targets when comparing CAF high with low group in entire TCGA cohorts, GS, CIN and MSI tumor. **B** Differential protein targets that are associated with overall survival; **C** Differential protein targets that are associated with progression-free survival

We then did the same comparison as RNA-seq section using TCGA RPRA data to search the proteomic variation of gastric cancer among samples with different fibroblast in entire TCGA cohort, GS, CIN and MSI subtype tumor, respectively. Since there are limited peptides presented in RPRA data and most targets of which the expressional change are statistically significant varied in relatively subtle scale (|logFC|< 1) compared with RNA-seq data, we regarded these targets as differentially expressed proteins as long as their P value are < 0.05. 38 hits visualized in Fig. 5A are targets that are differentially expressed in at least three independent analysis (Fig. 5A). Among these targets, MYH11, FN1, COL6A1 markers of CAF subpopulation determined by single-cell sequencing [34] are upregulated in high CAF group. Consistent with DEG in RNA-seq data, EMT marker CDH1 (E-cadherin) is downregulated, and TGF-beta/BMP pathway protein ACVRL1 [35] is increased. Proteins involved in DNA damage response and repair including MSH2, MSH6, two mismatch repair proteins and CHEK2, XRCC5 [14] also witness slight decreasing. Here, we also observed stronger expression of several apoptosis-associated proteins: BIRC2(cIAP1), CASP7(Casepase7) BCL2L11 and DIABLO in low CAF group. As a fatty acid synthesis enzyme, ACACA is more abundant in low CAF samples. What motivated us to reemphasize the importance of P13K/AKT pathway is Rictor, the core protein of mTOR2 complex who is responsible for AKT phosphorylated activation at serine 473 (Ser473) has remarkably higher expression level in high CAF samples [36]. However, mRNA expression is roughly the same implying high CAF fibroblast microenvironment rises Rictor expression by translational or post-translational regulation.

We examined the prognostic performance of these protein targets by survival analysis. Several upregulated proteins such as MYH11, RICTOR and ACVRL1 are bound to aggregated overall survival (Fig. 5B). RICTOR, CAV1 expression also indicates disease progression (Fig. 5C). Several proteins expressed stronger in low CAF samples are associated with better survival, especially CLDN7.

Similarly, we checked the shift of miRNA expressional profile in TCGA data. More than 100 mature miRNA strand varied with fibroblast (Additional file 7: Fig. S3A). Among these miRNAs, the expression of miR-8/ miR-200 family [37] microRNAs including mir-200, miR-141,miR-429 (Additional file 7: Fig. S3B), which exert tumor suppressive function are mitigated suggesting samples with higher CAF gathering may acquire a more aggressive phenotype.

### CAF infiltration and immune compromised microenvironment

**Fig. 6:**
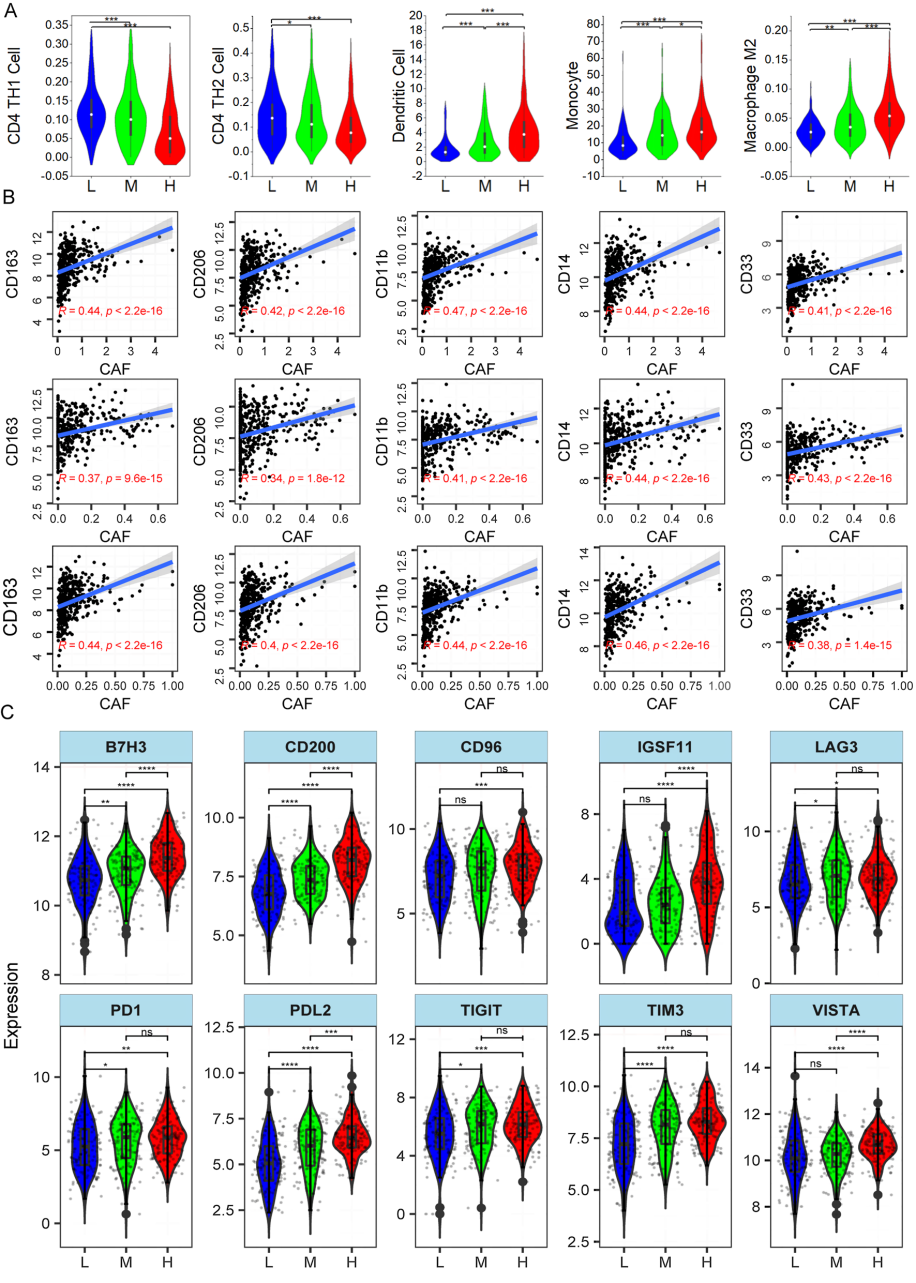
CAF infiltration and immune-suppressive micro-environment. **A** Relationship between CAF and Th1, Th2 CD4 cell, dendritic, monocyte and M2 macrophage; **B** CAF infiltration (in the top row, CAF was estimated by MCP-COUNTER; medium row, CAF was estimated by XCELL; bottom row, CAF was estimated by EPIC) and expression of M2 markers (CD163, CD206) and MDSC markers (CD11b, CD14, CD33). **C** Differential expression of immune checkpoint

To browse the immune-environment transformation brought by CAF infiltration in gastric cancer, we investigated connection between CAF and immune cell infiltration. Immune cells in TCGA were estimated by multiple approaching via TIMER2.0 online tool as described in method. Th1 and Th2, two CD4(+) T cell lineages mediated antitumor immune [38, 39] display higher estimated score in low CAF group than high CAF samples, while M2 macrophage, monocytes and dendritic cell are elevated in high CAF group(Fig. 6A). We re-validate this in GSE15459 cohorts by correlation analysis. As expected, CAF exhibits negative correlation with Th1 and Th2 cell. In contrast, positive correlation with M2 macrophage, monocyte and dendritic are detected (Additional file 8: Fig. S4). It’s widely accepted that M2 macrophages in cancer can be differentiated from monocytic myeloid-derived suppressor cells (m-MDSC), and another fortune of m-MDSC is inf-DC, a dendritic population [40]. Thus, to further validate CAF’s relationship to M2 macrophage and figure out whether MDSCs increases with fibroblasts, we analyzed the correlation between CAF and several well-defined markers for M2 macrophage and MDSC. Fortunately, CD163 and CD206, canonical marker for M2 macrophages [41] co-vary with fibroblasts quantified by three methods. CD11b, CD14, CD33, surface markers for MDSCs [42] are positively associated with CAFs. These results suggest CAF flooded gastric cancer microenvironment is accompanied with M2 macrophages and MDSCs expansion.

Finally, we reviewed the expression of suppressive immune checkpoints in gastric cancer. In general, ten checkpoints B7H3, CD200, CD96, IGSF11, LAG3, PD-1, PD-L2, TIGIT, TIM3, and VISTA increase with fibroblast as expression of all these ten checkpoints is higher in high CAF group (Fig. 6C). We also compared the immuno- suppressive modulators [43] and earned the same trend (Additional file 9: Fig. S5). These results promoted us concluding that CAF infiltration in gastric cancer is link to an immuno-suppressive tumor microenvironment.

### Prognostic risk model based on TGF-beta, RTKs and Hedgehog ligands

In our transcriptomics result, we mentioned PI3K/AKT, TGF-beta and Hedgehog Pathways are involved in CAF deeply infiltrated gastric cancer. All these three pathways mediate signaling transduction from extracellular secretory ligands to intra-cellular biological activity regulation. Amid highly complicated and frequent cell communication between tumor cells and stroma cells such as fibroblast and as well as immune cells, extracellular ligands are critical for the phenotypical plasticity of each type of cell. PI3K-AKT activation is coupled to receptor tyrosine kinases (RTKs) which hold dozens of ligands such as VEGF, PDGFs and FGFs [44, 45]. Thus, we seek to construct prognostic model with ligands for RTKs, TGF-beta and Hedgehog pathways (Additional file 4).

**Fig. 7:**
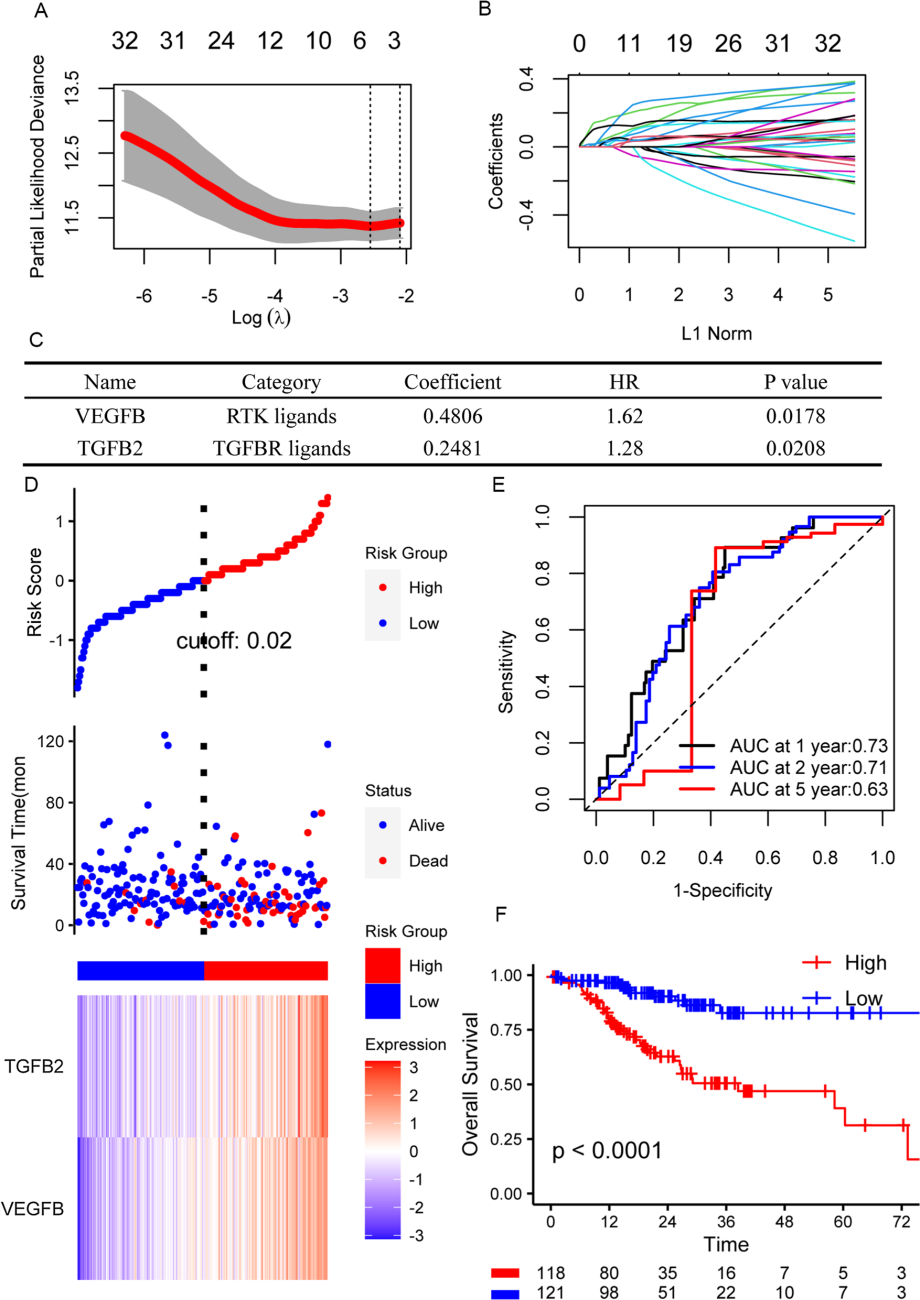
Construction of overall survival risk model based on 36 ligands for TGF-beta, Hedgehog pathways and RTKs. **A**, **B** Screening of candidate ligands for risk model from 36 ligands that are differentially expressed and associated with overall survival. **A** Shows cross-validation of the lasso COX model to get the optimal lambda value (minimum). **B** Shows the lasso COX regression coefficients of 36 ligands at different lambda values. **C** The coefficient of the risk sore model by multivariate COX regression. **D** Risk score and expression of TGFB2 and VEGFB for each individual in TCGA cohort. **E** ROC curves of predicted survival at 1 year, 2 year and 5 year time point. **F** Kaplan–Meier plot of low and high risk group

We tested the prognostic role of ligands that exhibit differentially expression at mRNA level in TCGA cohorts. Among the 69 differentially expressed ligands, 36 ligands are associated with overall survival by univariate COX model. We then utilized lasso cox for further screening and four ligands VEGFB, TGFB2, FGF14 and ANGPTL1 were in the lasso model (Fig. 7A, B). Therefore, we include these four ligands in our stepwise multi-variate COX analysis. Finally, a risk model consisted of TGFB2 and VEGFB were built with C-index to be 0.69 (Fig. 7C). Risk score at 0.02 were chosen as the cutoff value to define high and low risk group (Fig. 7D). The dead events are majorly distributed in high risk group with higher expressed TGFB2 and VEGFB. The AUC of the model for 1 years, 2 years and 5 years predicted survival are 073, 0.71, 0.62, respectively (Fig. 7E). The overall survival of high risk group is significantly shorter than low risk group (Fig. 7F).

**Fig. 8:**
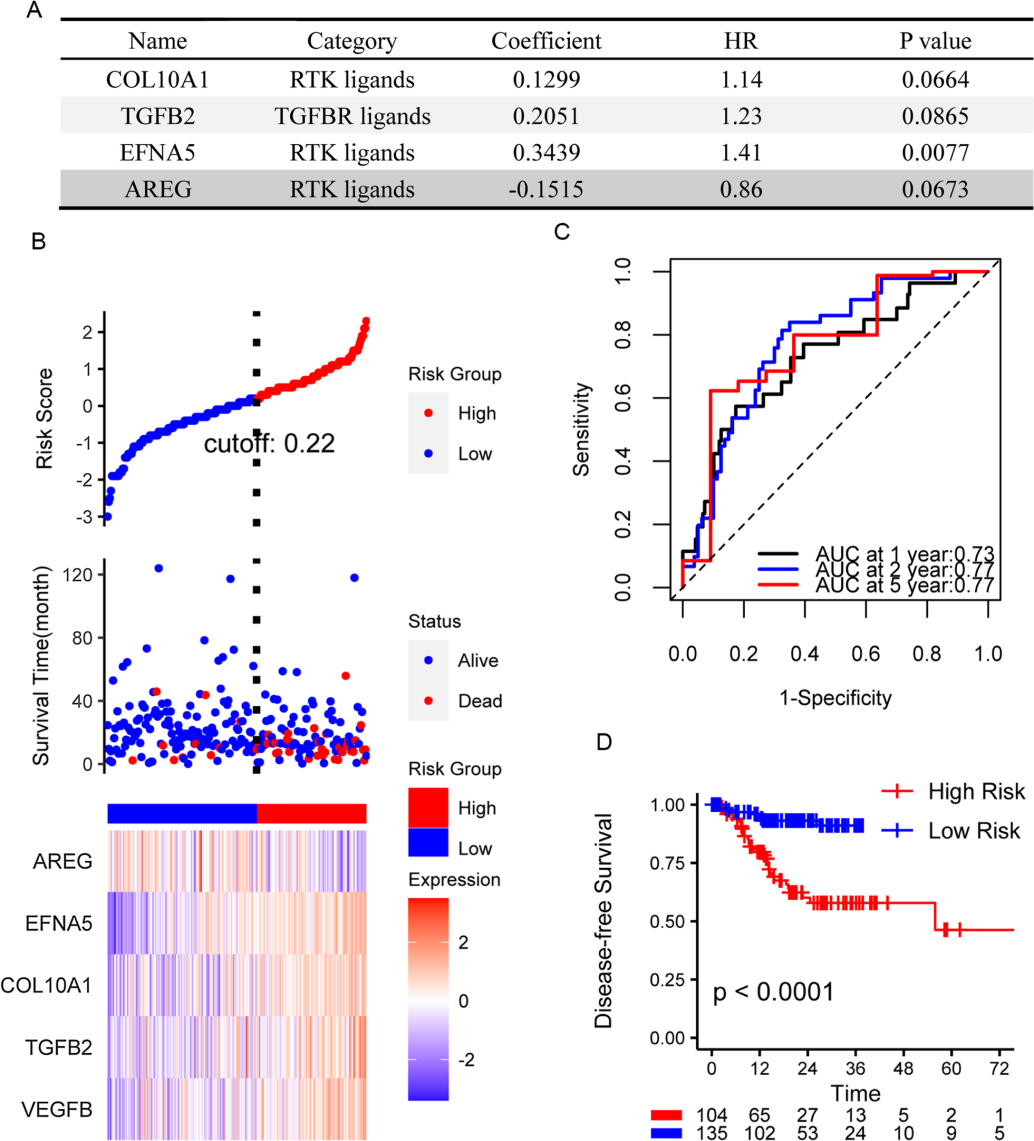
Risk model for disease-free survival. **A** Coefficients of the risk sore model by multivariate COX regression and the ligands in the model. **B** Risk score and expression of AREG, EFNA5, COL1OA1, TGFB2 and VEGFB for each individual in TCGA cohort. **C** ROC curve of predicted survival at 1 year, 2 year and 5 year time point. **D** Kaplan–Meier plot of low and high risk group

By the same workflow, we received a four gene risk score model to predict disease-free survival (Fig. 8A) in which TGFB2, a TGFB receptor ligand, and COL10A1, EFNA5 AREG, three RTK ligands were included. C-index of the model can be as high as 0.73. AUC for 1 years, 2 years and 5 years disease-free survival are 0.73, 0.77 and 0.77 (Fig. 8C). The risk score can recognize the patients with poorer disease-free survival explicitly (Fig. 8B, D).

We validated the two models in GSE62254 cohorts. Despite the AUC of ROC are not satisfying (Additional file 10: Fig. S6A, C), the Kaplan–Meier plots demonstrate that higher risk score can separate the patients with poorer survival well (Additional file 10: Fig. S6B, D). We also confirmed the performance of the score system in GSE15459 and GSE84437 (Additional file 10: Fig. S6E, F). In summary, ligands of TGFB receptor and RTKs based risk score system can efficiently predict the survival of gastric cancer patients.

## Discussion

Recent decade, surging studies announce CAF’s tumor booster role via affecting stromal–epithelial interactions, immunity, angiogenesis and ECM re-modeling in solid tumors such as pancreatic and colorectal cancer [46]. As a type cancer frequently associated with abundant fibrosis [47], CAF in gastric cancer should be given more concern. However, the current research is very limited regarding global function of CAF in gastric cancer.

In this study, we inspected the link of CAF infiltration to subtypes and disease stages. To our knowledge, we are the first to point out CAF infiltration is higher in DGC and GS tumor that both are poorer survival subtypes in their corresponding classification system [25, 28]. This difference might be explained by the overlapping of DGC and GS with scirrhous gastric cancer characterized as rapid expansion and invasion of poorly differentiated or signet‐ring cancer cells with extremely surrounding fibrosis [48]. Our result also reveals CAF infiltration is generally higher in stage III and IV patients implying the involvement of CAF in disease progression. Although increasing studies revealed the role of CAF in cancer by experimental study, CAF’s prognostic indicative role in gastric cancer is seldom conducted. In presented study, we estimated the outcome of gastric cancer brought by high CAF infiltration. The result suggests CAF is a independent prognostic factor.

We uncovered wide transcriptomics and pathways alteration in CAF high infiltrated tumor by DEG and GSEA. Among these, PI3K/AKT, TGFB and Hedgehog pathway are potential key pathways. We used ligands for TGFB, Hedgehog pathways and RTKs, upstream of PI3K/AKT [49] to construct survival risk model. TGFB2 and VEGFB were selected as risk predictors for both overall and disease-free survival highlighting the role of TGFB and PI3K/AKT pathway in gastric cancer. Meanwhile, it’s worth studying that whether patients with high CAF infiltration in tumor tissue could benefit more from PI3K-AKT and TGF-beta pathway inhibitive treatment in the future.

Interestingly, gene sets involved in DNA damage response and repair are enriched in tumors with fewer CAF infiltration. Comparison of MSI, aneuploidy and HRD score in high, medium and low CAF group proposed us to consider tumors lacking CAF infiltration might undergo more sever DNA damage and genomic alteration. Another evidence for this is the increased infiltration of CAF in genomically stabe gastric cancer compared with instable tumor (MSI and CIN). Actually, it was reported that CAF promotes esophageal squamous cell carcinoma DNA repair via upregulating a lncRNA named DNM3OS [50]. In our study, even though the phenomenon steadily occurs in different subgroups, it’s difficult to conclude who is the cause and who is the consequence in this inverse relationship. To be concrete, CAF in microenvironment might help tumor getting rid of harmful DNA damage and on the contrary, the existence of DNA damage might prevent CAF recruitment or activation. As a pure observational study, we are not able to guarantee which situation it represents.

With the prosperity of immune therapy, some researchers try to decipher the sophisticated interaction of CAF with immune cell. CAF function in the recruitment activation of tumor promotive immune cell like MDSC and TAM have been discovered in other types of tumor [46]. Here, we validated the positive correlation of CAF to MDSC markers and M2 macrophages. We found ten immune checkpoints have higher expression in gastric cancer with higher CAF infiltration as well, indirectly supporting the notion that CAF is associated with immune-suppressive micro-environment.

However, we have our limitations in this study. First of all, we employed pure bioinformatic method to estimate CAF as well as immune cells. How far it’s from real-world should be carefully treated. To avoid the bias CAF estimation method might bring, we transformed CAF score into ordinal categorical variable and got a consensus high or low CAF group to perform our later analysis. The computational algorithms involved in this study including, MCPCOUNTER, XCELL and EPIC quantify cell types abundance by either cell signature based GSEA or deconvolution of cell mixtures from gene expression matrix [21], and all the methods allow comparisons of the same cell type between samples. In addition, multiple datasets were used in this study to ensure the robustness of our results which still can provide referential value for understanding clinical and tumor biological significance of CAF.

Secondly, it’s difficult to dissect expression profiles of tumor cell itself, stroma cell component and immune cell from bulk tumor tissue. In this case, the cellular localization of transcriptomic and pathway alteration needs to be elucidated by more experimental study. What need to be noticed is CD11b, CD14 and CD33 are not definitely specific markers for MDSC, the connection between CAF and MDSC should be carefully interpreted, and more work defining MDSC with gold standard such as flowcytometry have to be done in the future. Lastly, we roughly reviewed the connection of CAF to clinical characteristics, prognosis and immune micro-environment without considering CAF subtype and time-spatial heterogeneity. For the reason of CAF molecular and functional heterogeneity, none of currently used markers are exclusively expressed by all CAF populations [51, 52], posing a challenge for us to understand CAF panorama. Hopefully, single cell sequencing (scRNA-seq) technology may be an excellent strategy to make a breakthrough [52]. CAF subtypes in breast and pancreatic cancer identified by scRNA-seq have been reported [53, 54].We look forward to the gap in gastric cancer filed to be filled soon.

## Conclusion

CAF infiltration is more sever in DGC, GS tumor and stage III/IV. CAF infiltration is associated with immune-suppressive microenvironment and worse survival for gastric cancer. In summary, CAF infiltration engages in the acquirement of aggressive cancer phenotype. Simultaneously, TGFB2, VEGFB, COL10A1, ERG1 and EFNA5 composed risk model is a promising tool for assessment of gastric cancer survival.

## Supplementary Information

**Additional file 1**: Overall survival of gastric cancer in five cohorts, CAF stratified by MCPCOUNTER, XCELL, EPIC respectively.

**Additional file 2**: GSEA results in entire TCGA cohort, MSI, CIN and GS subtype.

**Additional file 3**: GSEA results in GSE62254 cohort.

**Additional file 4**: TGF-beta pathway, Hedgehog pathway and RTKs ligands list.

**Additional file 5**: Fig. S1 A. CAF infiltration in different Lauren subtypes. The proportion of low (L) medium (M) and high (H) infiltration in each type B. CAF infiltration in stage III/IV samples across different molecular subtypes.

**Additional file 6**: Fig. S2 Kaplan–Meier plots of GSE62254, GSE26901, GSE 26,253 cohorts grouped by CAF infiltration.

**Additional file 7**: Fig. 3 Differentially expressed miRNA. A heatmap displaying expression of miRNA in each TCGA samples; B representative miRNA families that are upregulated or downregulated in high CAF group.

**Additional file 8**: Fig. 4 CAF and immune cell correlation heatmap in GSE15459 cohort.

**Additional file 9**: Fig. 5 immuno-suppressive modulators expression in TCGA cohort.

**Additional file 10**: Fig. 6 Validation of risk score model in GSE62254, GSE15459 and GSE84437 cohorts. A-B evaluating the risk model for overall survival in GSE62254 cohort by ROC and Kaplan–Meier plot; C-D evaluating the risk model for disease-free survival in GSE62254 cohort; E–F Kaplan–Meier plot of GSE15459 and GSE84437 cohorts grouped by risk score for overall survival.

## Data Availability

TCGA RNA-seq and clinical information data is deposited in XENA UCSC (https://xenabrowser.net/datapages/), RPRA data is from cBioPortal (http://www.cbioportal.org/). All the GEO datasets including GSE15459, GSE62254, GSE84437, GSE26901, GSE26253 can be downloaded from Gene Expression Omnibus database of NCBI (https://www.ncbi.nlm.nih.gov/gds/).
