# Supplementary figures and images for: Cancer-associated fibroblast infiltration in gastric cancer: the discrepancy in subtypes pathways and immunosuppression
Source: J Transl Med. 2021 Jul 31;19:325. doi: 10.1186/s12967-021-03012-z (PMC8325313; doi:10.1186/s12967-021-03012-z)

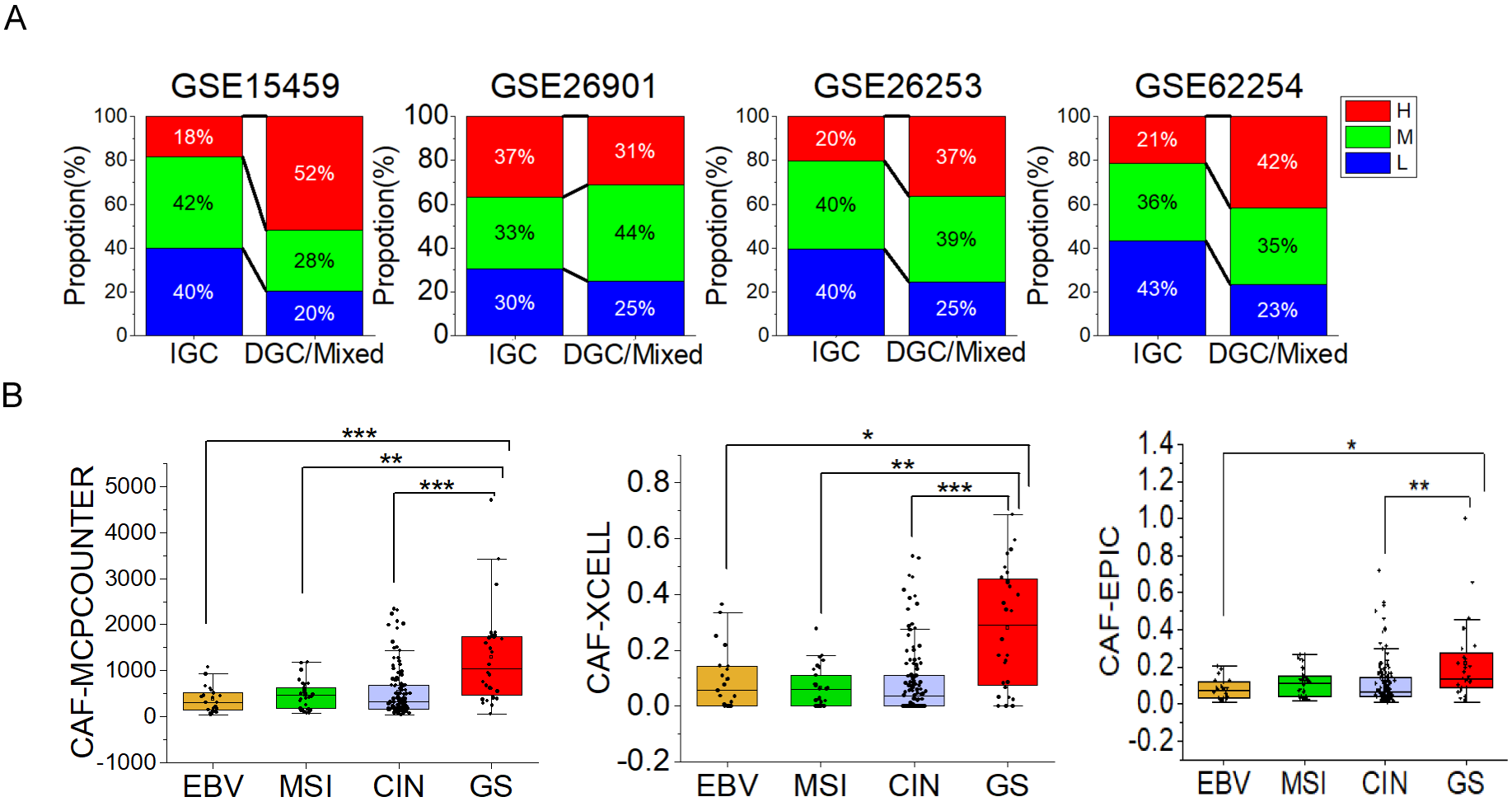

Supplement: Supplementary file 5 — Additional file 5: Fig. S1 A. CAF infiltration in different Lauren subtypes. The proportion of low (L) medium (M) and high (H) infiltration in each type B. CAF infiltration in stage III/IV samples across different molecular subtypes. [file 12967_2021_3012_MOESM5_ESM.tif]

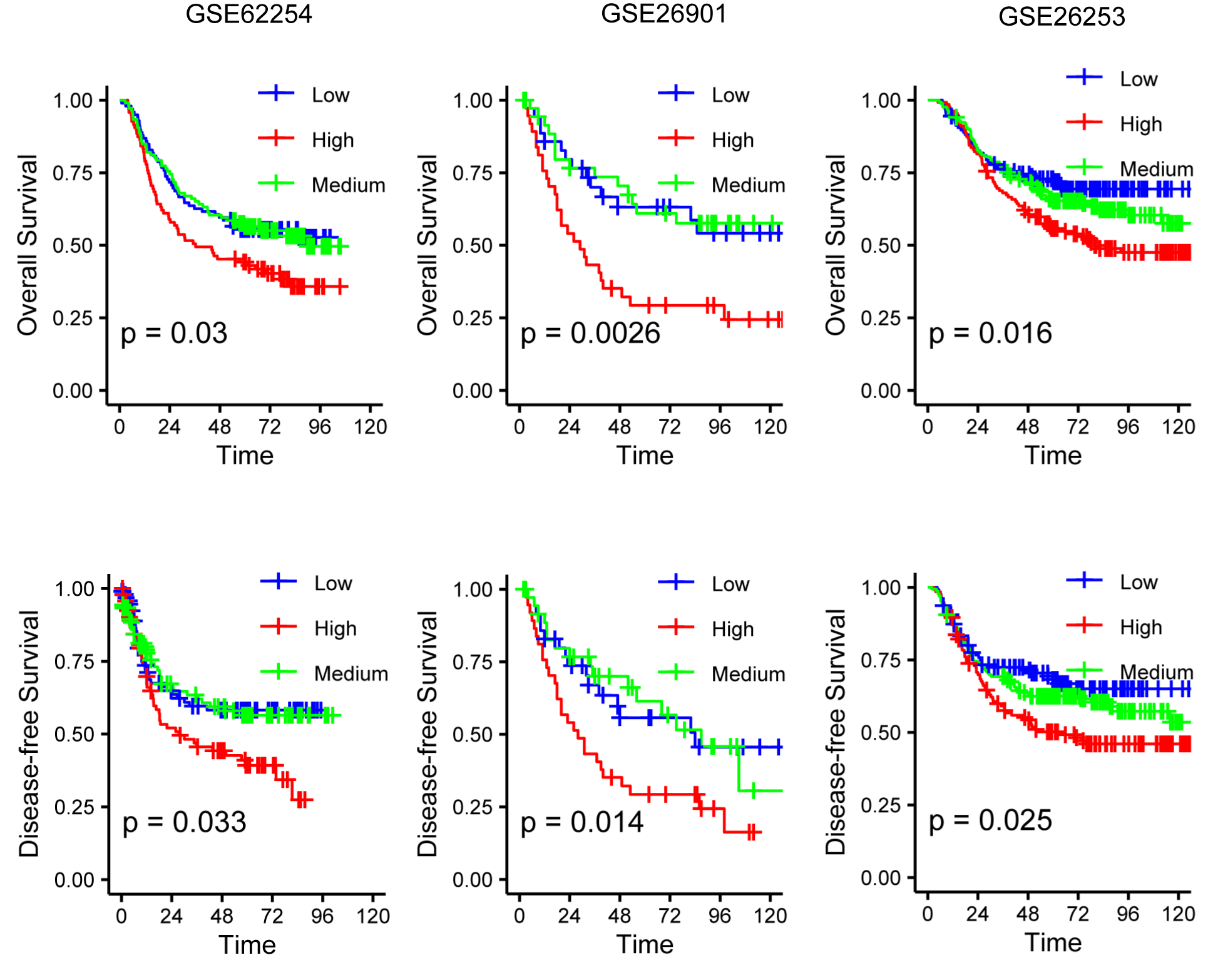

Supplement: Supplementary file 6 — Additional file 6: Fig. S2 Kaplan–Meier plots of GSE62254, GSE26901, GSE 26,253 cohorts grouped by CAF infiltration. [file 12967_2021_3012_MOESM6_ESM.tif]

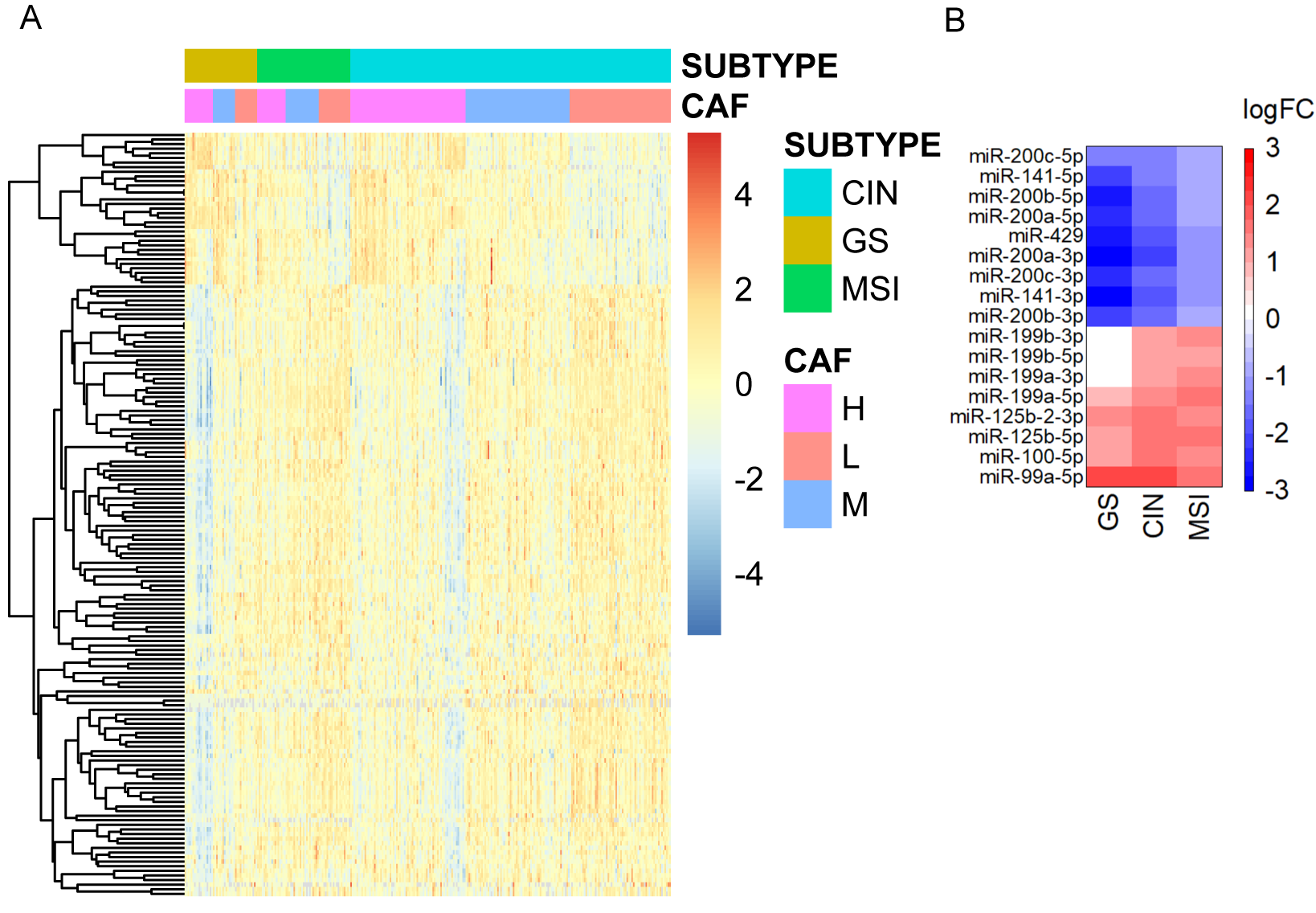

Supplement: Supplementary file 7 — Additional file 7: Fig. 3 Differentially expressed miRNA. A heatmap displaying expression of miRNA in each TCGA samples; B representative miRNA families that are upregulated or downregulated in high CAF group. [file 12967_2021_3012_MOESM7_ESM.tif]

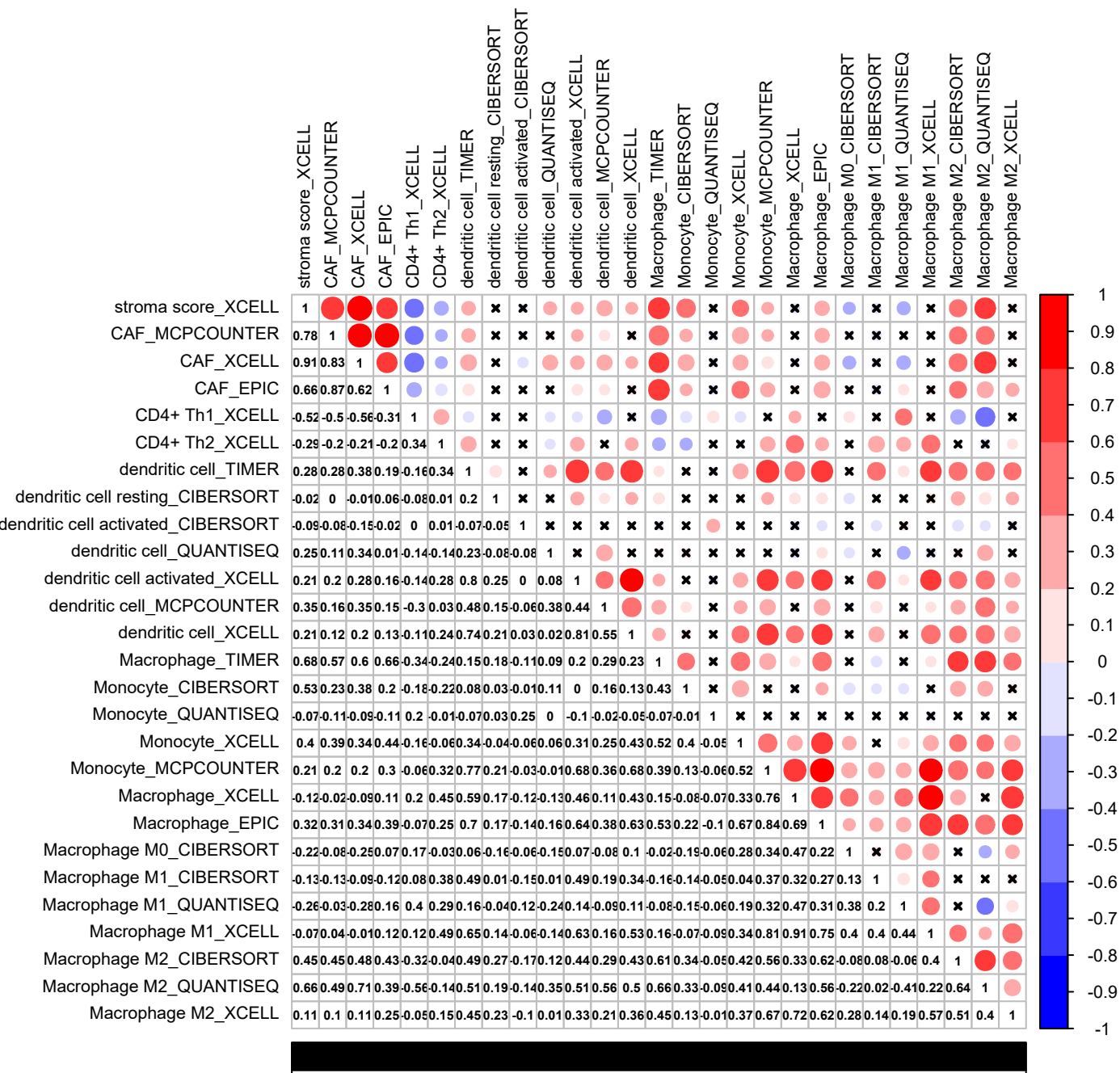

Supplement: Supplementary file 8 — Additional file 8: Fig. 4 CAF and immune cell correlation heatmap in GSE15459 cohort. [file 12967_2021_3012_MOESM8_ESM.pdf]

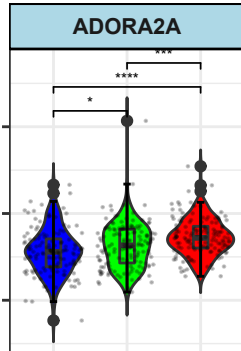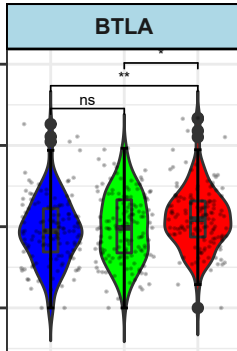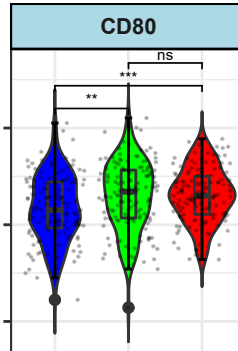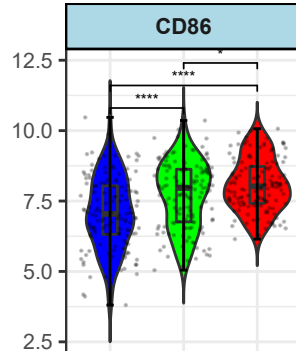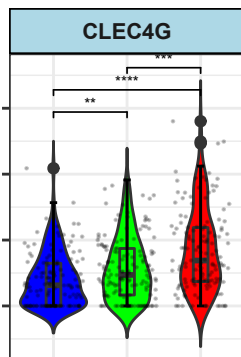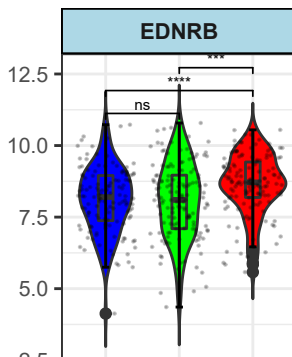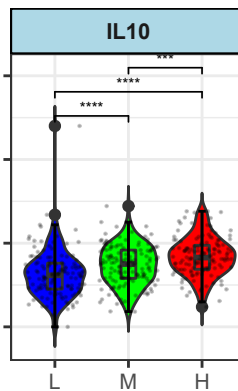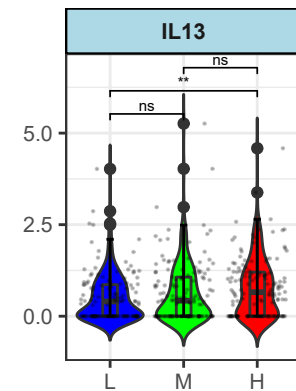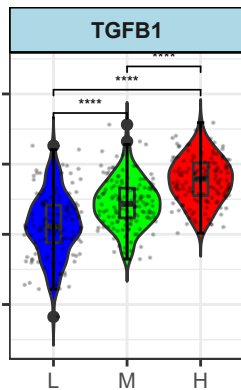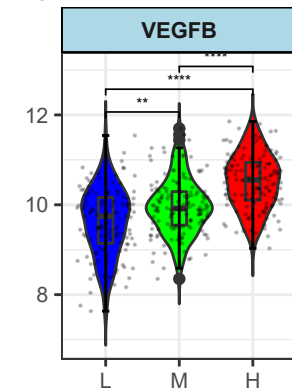

Supplement: Supplementary file 9 — Additional file 9: Fig. 5 immuno-suppressive modulators expression in TCGA cohort. [file 12967_2021_3012_MOESM9_ESM.pdf]

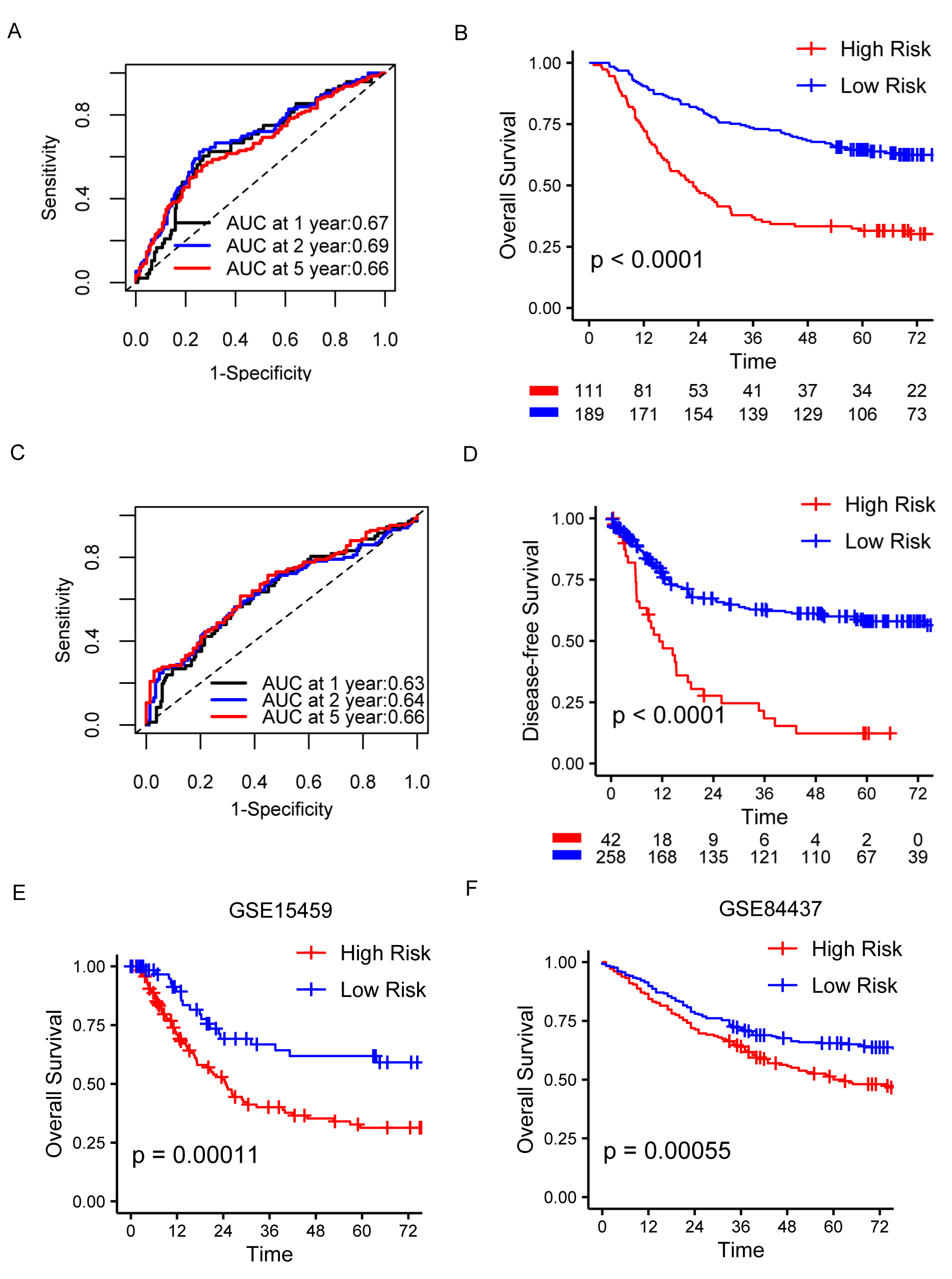

Supplement: Supplementary file 10 — Additional file 10: Fig. 6 Validation of risk score model in GSE62254, GSE15459 and GSE84437 cohorts. A-B evaluating the risk model for overall survival in GSE62254 cohort by ROC and Kaplan–Meier plot; C-D evaluating the risk model for disease-free survival in GSE62254 cohort; E–F Kaplan–Meier plot of GSE15459 and GSE84437 cohorts grouped by risk score for overall survival. [file 12967_2021_3012_MOESM10_ESM.tif]
